# A Review of Combined Phosphodiesterase-5-Inhibitors and α-Blockers versus Phosphodiesterase-5-Inhibitors Alone for Lower Urinary Tract Symptoms due to Benign Prostatic Hyperplasia

**DOI:** 10.1080/2090598X.2023.2220627

**Published:** 2023-06-13

**Authors:** Elizabeth M Jackson, Prajit Khooblall, Scott D Lundy, Petar Bajic

**Affiliations:** a School of Medicine, Case Western Reserve University, Cleveland, USA; b Cleveland Clinic Glickman Urological and Kidney Institute, Cleveland, USA

**Keywords:** PDE-5 inhibitors, LUTS/BPH, ED, tadalafil, quality of life

## Abstract

Guidelines from the American Urological Association (AUA) and the European Association of Urology (EAU) present conflicting recommendations regarding combination therapy of phosphodiesterase 5 inhibitors (PDE5is) with α-blockers to treat benign prostatic hyperplasia (BPH) with lower urinary tract symptoms (LUTS). Use of PDE5is is widespread in the population of patients with LUTS/BPH. In this scoping review, we examine the evidence regarding the safety and efficacy of combined PDE5is and α-blockers compared to PDE5i medications alone. A search was conducted using PubMed, Cochrane, and Web of Science to identify manuscripts discussing the safety of PDE5i and α-blockers in combination or comparing this combination to PDE5is alone in the treatment of LUTS/BPH. Study designs, data, and conclusions were qualitatively analyzed. Combination therapy was found to be safe across all studies; importantly, no evidence documents increased risk of hypotension. Most studies reported added improvement in symptom and quality of life scores compared to PDE5i alone, with additional International Prostate Symptom Score (IPSS) change ranging from −1.30 to −8.50 and IPSS quality of life score change ranging from −0.15 to −1.50. Objective metrics such as postvoid residual volumes and maximum flow rate were inconsistently reported. Taken together, the current body of data suggests that combining PDE5i α-blocker therapy is safe and that there are opportunities for additional symptomatic improvement, though it should be utilized for select patients. Situations with particular utility could include patients with comorbid erectile dysfunction or without sufficient improvement on monotherapy.

## HIGHLIGHTS

KEY POINTS

- combination therapy with PDE5i and α-blockers is more effective than PDE5i medications alone for lowering IPSS
- combination therapy with PDE5i and α-blockers is not associated with a significantly greater number of adverse events than PDE5i medications alone
- the improvements seen in IPSS with combination therapy compared to PDE5i alone may or may not reach the threshold of clinical significance
- PDE5i and α-blocker combination therapy should be considered a safe regimen that can be used in appropriate clinical situations, like for patients with comorbid ED and those who do not achieve sufficient control of symptoms with a daily PDE5i alone

## Purpose of review

From their initial discovery and identification as a treatment for erectile dysfunction (ED), phosphodiesterase-5 inhibitors (PDE5i) have been widely studied for safety, tolerability, ease of use, and efficacy [1,2]. PDE5i medications work by inhibiting the action of phosphodiesterase type 5, which delays breakdown of cGMP, allowing relaxation and inflow of blood into the corpus cavernosum. In more recent years, there has been an expansion of the indications for this drug class, particularly with regard to lower urinary tract symptoms (LUTS) due to benign prostatic hyperplasia (BPH). These two constellations of symptoms are highly comorbid. In men suffering from ED, the prevalence of LUTS is above 70% [3]. In men with moderate-to-severe LUTS, the prevalence of sexual dysfunction including ED has been shown to be increased compared to general population rates [4,5]. The Cologne Male Survey results showed that LUTS is an independent risk factor for the development of ED with an odds ratio of 2.11 [3]. Tadalafil was approved by the FDA for the treatment of LUTS/BPH in 2011, making it the only PDE5i drug currently approved for this indication [6]. Due to their systemic vasodilatory effects, PDE5i medications should not be taken in those who are at risk of developing severe hypotension [7–10]. This includes patients with certain cardiac or blood pressure issues, as well as those who are taking nitrates or some other classes of medications. PDE5i medications are also contraindicated in patients with certain ophthalmologic issues [1,2,7,9–11].

Preceding PDE5is by many years, α-blockers have been the long-time mainstay therapy for treating LUTS/BPH. Both the American Urological Association (AUA) and the European Association of Urology (EAU) guidelines strongly recommend offering an α-blocker to patients with moderate-to-severe LUTS/BPH in patients for whom lifestyle modifications are insufficient. Additionally, the AUA guidelines specifically list this class as first-line medical therapy [12–14]. This medication class inhibits the effects of norepinephrine on smooth muscle in the prostate and bladder neck, helping to relieve obstruction by reducing muscle tone. This smooth muscle relaxation also causes a reduction in vascular tone and can cause side effects of orthostatic hypotension. α-blockers have been associated with a statistically significant increase in the odds of having a vascular adverse event, with an odds ratio of 2.54 [15]. The α-blockers with the most prominent vasodilatory effects are doxazosin and terazosin, with this effect less common for alfuzosin and tamsulosin [14].

Contemporary research for LUTS/BPH has also investigated the combination of existing medications to assess additional benefits for patients. As two of the most effective medications alone, PDE5i drugs and α-blockers have been studied in conjunction. However, clinicians have been wary of using this combination in practice due to the theoretical risks of hypotension and unknown synergy associated with both medication classes.

The AUA and the EAU both provide recommendations on the treatment of LUTS/BPH based on available evidence for safety and efficacy. Though they both recommend α-blocker monotherapy for bothersome moderate-to-severe LUTS and acknowledge the role of PDE5i medication in patients with or without ED, they differ in their preferred treatment and discussion of combination therapy [12,14]. The AUA guidelines prefer α-blockers as a first-line therapy, whereas the EAU equally recommends PDE5i medications and α-blockers. For combination therapies involving PDE5i medications, the EAU gives no official recommendations but acknowledges initial data that show combined PDE5i and α-blocker is well tolerated and effective. The AUA recommendations state that clinicians should not offer α-blockers and daily tadalafil concurrently, citing Grade C evidence that it offers no additional improvement over either monotherapy [2,14]. The discrepancy in stated guidelines about PDE5i and α-blocker combination therapy indicates that the body of evidence is complex and that reviewing available literature can lead to interpretations with varying conclusions.

In summary, the current recommendations for LUTS/BPH do not provide clear, consistent guidance on whether PDE5i drugs and α-blockers can be useful in combination or as add-on therapies. There remains a need to provide an updated analysis of the body of research evidence to ensure that both medications are being best used for this indication, especially in populations that may benefit from PDE5i drugs for ED. PDE5i medications are approved to treat both conditions, though α-blockers are often the initial prescribed medication for LUTS/BPH. Additionally, several PDE5i medications including sildenafil, tadalafil, and vardenafil are available as generics at low cost, providing an opportunity to use this drug class to treat multiple conditions at low financial burden and without additional risk of side effects [6,16]. Maximizing the evidence-supported uses for this drug class could improve patient adherence and outcomes and simplify multidrug regimens for patients with multiple urologic conditions. This scoping review analyzed literature comparing PDE5i medications alone with PDE5i medications and α-blockers for LUTS/BPH to better understand the differing AUA and EAU guidelines and to provide a focused review to help guide treatment choices for clinicians.

## Methods

### Search strategy

The literature search was conducted in November 2021 using PubMed, Cochrane, and Web of Science to find relevant papers and resulted clinical trials. Each search used the following set of terms: [(PDE5 inhibitors) OR (PDE5i) OR (Phosphodiesterase 5 inhibitors)] AND [(BPH) OR (benign prostatic hyperplasia) OR (LUTS)] AND [(quality of life) OR (IPSS) OR (international prostate symptom score)] AND [efficacy]. The search was restricted to papers published between 1 January 1993 and 11 January 2021. The full list of results from each database was uploaded to the Covidence tool for review.

### Inclusion and exclusion criteria

The paper selection process was performed using Covidence. Two reviewers (EJ and SL) assessed the papers at each stage and a third reviewer (PB) reviewed any discrepancies. The initial title and abstract review excluded studies that were duplicate, irrelevant, or that did not fit inclusion criteria. The remaining papers underwent a full-text review to determine final inclusion or exclusion. Papers included in the scoping review must have been identified according to the described search in at least one electronic database. Eligible studies included clinical trials, randomized controlled trials, cross-sectional studies, observational studies, and meta-analyses. Both prospective and retrospective study designs were included. To be included in discussions of treatment efficacy, papers must have assessed PDE5i medications alone compared to PDE5i medications with α-blockers for LUTS/BPH. To be included in discussions of safety, papers must have assessed the combination of PDE5i medications with α-blockers for LUTS/BPH. Excluded papers were those that did not meet inclusion criteria, were not available in English, only discussed drugs not widely available in the United States (e.g. mirodenafil, udenafil, and/or lodenafil), were performed in animal models or on specific patient subsets, or had treatment groups of less than 25 patients.

## Findings

### Study selection/data extraction

**Figure 1.**
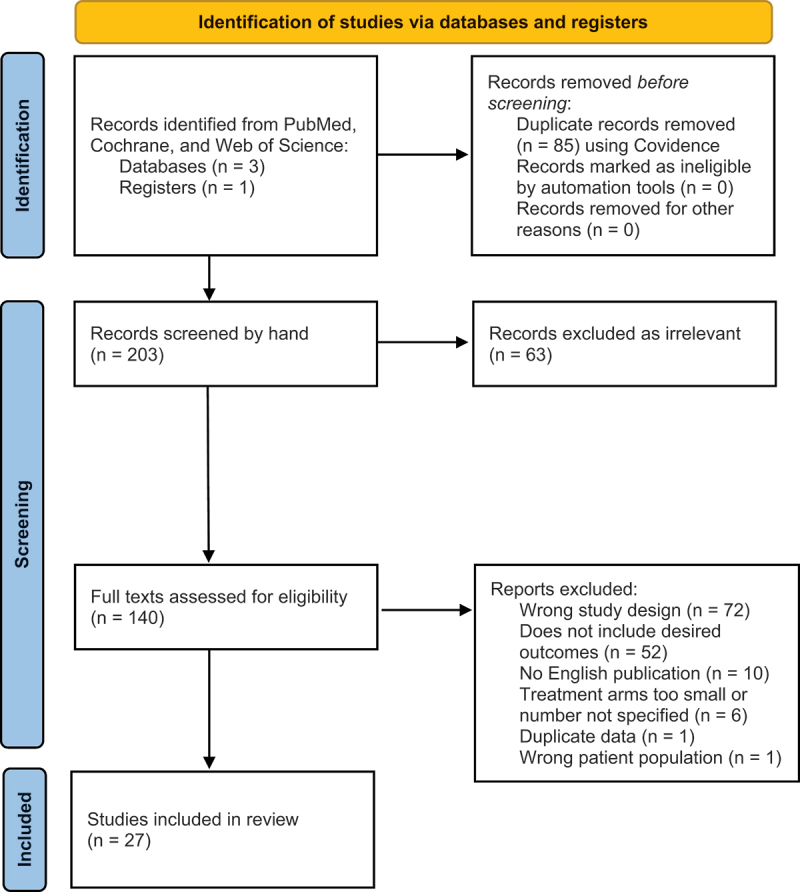
PRISMA diagram for literature acquisition and selection process.

Our search identified 288 total citations. Title and abstract screening excluded 148, leaving 140 manuscripts for full-text review. Based on the inclusion and exclusion criteria, 27 studies were included for the safety review and 13 papers for the treatment efficacy review. The data extracted included the study aim, population, interventions, and any reported outcomes, including objective measures, subjective and quality of life measures, and safety data. The study selection process, including reasons for study exclusion, is summarized in Figure 1.

### Study characteristics

The studies included were comprised of 10 randomized trials, 4 nonrandomized trials, and 12 meta-analyses. They were published from 2011 to 2020 and represented a variety of countries. The findings from these studies can be found in Table 1.

**Table 1.** Results of literature review for studies included in the safety review and in the efficacy review.

Safety Review				
Author	Study Design	Number of Participants	Intervention	Findings
Chen 2020	Meta-analysis	1173	α-blocker ± vardenafil or sildenafil	AE rate over study period: **significantly increased with combination therapy (18.9% vs 7.2%)** Dropout rate: no significant difference with combination therapy (4.38% vs 2.63%) AEs in combination group: headache, dizziness
Dahm 2017	Systematic Review and Meta-analysis	565	Tamsulosin ± PDE5i	AE rate over study period: insufficient data Dropout rate: insufficient data AEs in combination group: headache, flushing, stomach pain (with tamsulosin + vardenafil) No severe adverse effects
Gacci 2012	Randomized controlled trial	60	Tamsulosin ± vardenafil	AE rate over study period: no significant difference with combination therapy (10% vs 6.9%) Dropout rate: 0% in combination therapy group, 3.33% in tamsulosin only group AEs in combination group: headache (3.33%), stomach pain (6.67%), fainting (3.33%) No severe adverse effects in either group
Gacci 2012 (2)	Meta-analysis	2749	PDE5i ± α-blocker	AE rate over study period: no significant difference with combination therapy Dropout rate: not compared AEs in combination group: headache, dyspepsia, dizziness
Gupta 2016	Non-randomized experimental study	60	Tadalafil + α-blocker	Dropout rate: not reported AEs: headache (13.33%), nasopharyngitis (8.33%), dyspepsia (5.0%)
Jin 2011	Randomized controlled trial	203	Sildenafil ± doxazosin	AE rate over study period: no significant difference with combination therapy (15.4% vs 14.6%) Dropout rate: 0% in both groups AEs in combination group: headache (5.4%), dizziness (4.8%), flushing (3.6%), palpitation (3.6%), dyspepsia (3.0%), diarrhea (2.4%), acratia (1.2%), abdominal pain (0.6%) No severe adverse effects in either group
Kallidonis 2020	Meta-analysis	not reported	PDE5i, α-blocker, combination	AE rate over study period: **significantly decreased with α-blocker monotherapy compared with combination therapy (RR 0.61)** Dropout rate: not reported AEs in combination group: nasopharyngitis, abdominal discomfort, dyspepsia, flushing, headache, ocular hyperemia, myalgia, dizziness, retrograde ejaculation, gastrointestinal disorders No severe adverse effects in any group
Kim 2017	Randomized controlled trial	432	Tadalafil ± tamsulosin	AE rate over study period: **significantly increased with combination therapy (14.11% vs 8.43%)** Dropout rate: not compared AEs in combination group: headache (5%), nasal congestion (3%), ocular hyperemia (3%) No severe adverse effects in either group
Kumar 2014	Randomized controlled trial	75	Tadalafil, alfuzosin, combination	AE rate over study period: not compared Dropout rate: 0% in all treatment groups AEs in combination group: headache (12%), dizziness (8%)
Lee 2012	Non-randomized experimental study	158	Tadalafil + α-blocker	AE rate over study period: 5.04% Dropout rate: 1.27% due to AEs AEs in combination group: facial flushing (3.36%), headache (1.46%), dizziness (1.46%) No significant change in baseline blood pressure and heart rate
Ma 2020	Meta-analysis	531	Tamsulosin ± PDE5i	AE rate over study period: of all combination therapies, lowest in sildenafil and tamsulosin group Dropout rate: not compared AEs in combination group: not included
Nagasubramanian 2020	Randomized controlled trial	140	Tamsulosin ± tadalafil	AE rate over study period: no significant difference with combination therapy Dropout rate: 1.4% in monotherapy group, 4.35% in combination group AEs in combination group: myalgia (7.25%), dyspepsia (2.90%) No serious adverse effects in either group
Ozturk 2012	Randomized controlled trial	100	Alfuzosin ± sildenafil	No safety data included
Safety Review				
Author	Study Design	Number of Participants	Intervention	Findings
Qiangzhao 2020	Meta-analysis	not reported	α-blocker ± PDE5i	No serious adverse effects in either group
Sebastianelli 2019	Non-Randomized experimental study	75	Tadalafil ± tamsulosin	AE rate over study period: no significant difference with combination therapy (22% vs 16%) Dropout rate: 0% in both groups AEs in combination group: headache (8%), back pain (6%), ejaculatory dysfunction (4%), dyspepsia (2%), dizziness (2%) No serious adverse effects in either group
Singh 2014	Randomized controlled trial	125	Tadalafil, tamsulosin, combination	AE rate over study period: not compared Dropout rate: 0% in tamsulosin group, 6.82% in tadalafil group, 2.27% in combination group AEs in combination group: dyspepsia (13.64%), flushing (4.55%), headache (4.55%), myalgia (4.55%) No serious adverse effects in any group
Sun 2018	Meta-analysis	1052	α-blocker, PDE5i, combination	AE rate over study period: **significantly increased with combination therapy compared to α-blocker (RR 3.40)**, no significant difference with combination therapy compared to PDE5i Dropout rate: not compared AEs in combination group: headache, dyspepsia
Kai Sun 2020	Meta-analysis	1144	α-blocker, PDE5i, combination	AE rate over study period: **significantly increased with combination therapy (OR 2.85 compared to tamsulosin alone, OR 1.47 compared to tadalafil alone)** Dropout rate: no significant difference between groups AEs in combination group: myalgia, back pain, headache, bone pain
Yi Sun 2020	Meta-analysis	2472	α-blocker, PDE5i, combination	AE rate over study period: **significantly increased with combination therapy compared to α-blockers alone (RR 2.25)**, no difference compared to PDE5i alone Dropout rate: not reported AEs in combination group: headache, flushing, dizziness, diarrhea, dyspepsia, hypotension
Takeda 2017	Randomized controlled trial	171	Tadalafil ± α-blocker	AE rate over study period: no significant difference with combination therapy (28.1% vs 24.2%) Dropout rate: 4.8% with combination therapy, 1.9% with monotherapy AEs in combination group: dizziness (1.2%), vertigo (0.6%), nausea (0.6%), hepatic function abnormal (0.6%), decreased appetite (0.6%), hypoesthesia (0.6%), urinary urgency (0.6%), urinary retention (0.6%), asthma (0.6%) No serious adverse effects in either group, including orthostatic hypotension Objective BP changes: Changes in SBP between groups not statistically significant **DBP decreased in combo therapy (−1.6 mmHg) – statistically significant, not clinically significant** **Pulse increased in combo therapy (1.4 bpm) – statistically significant, not clinically significant**
Urakami 2019	Randomized controlled trial	75	α-blocker + tadalafil or solifenacin	AE rate over study period: not reported Dropout rate: no significant difference between tadalafil add-on and solifenacin add-on groups (18% vs 32%) AEs in PDE5i combination group: stomach discomfort/nausea (7.9%), dizziness/vertigo (5.2%), headache (2.6%), blurred vision (2.6%), constipation (2.6%), stomatitis (2.6%), morning erections (2.6%), voiding difficulty (2.6%) No significant change in BP in either group or between groups
Wang 2014	Meta-analysis	29384	PDE5i, α-blocker, combination	AE rate over study period: higher incidence reported with combination therapies, no statistical comparison performed Dropout rate: not reported AEs in combination group: headache (5.36%), dizziness (4.76%), flushing (3.17%), dyspepsia (2.98%)
Wang 2015	Meta-analysis	not reported	PDE5i, α-blocker, combination	AE rate over study period: no significant difference between groups (15.1% vs 26.5% with PDE5i alone and 21.9% with α-blocker alone) Dropout rate: not reported AEs in combination group: headache (13.7%), myalgia (5.0%), dizziness (4.7%), flushing (3.2%), dyspepsia (3.9%)
Yan 2014	Meta-analysis	515	PDE5i, α-blocker, combination	No safety data included
Yoshida 2017	Non-randomized experimental study	103	Silodosin ± tadalafil	AE rate over study period: no significant difference between groups Dropout rate: no significant increase with combination therapy (3.8% vs 0%) AEs in combination group: headache (1.9%), palpitations (1.9%), peripheral edema (1.9%), dyspepsia (1.9%)
Safety Review				
Author	Study Design	Number of Participants	Intervention	Findings
Zhang 2019	Meta-analysis	855	α-blockers ± PDE5i	AE rate over study period: not reported Dropout rate: not reported AEs in combination group: dizziness (2.36%), flushing (8.60%), gastrointestinal disorders (4.36%), headache (15.27%), myalgia (6.41%), nasopharyngitis (7.44%) No serious adverse effects
Zhou 2019	Meta-analysis	621	Tadalafil ± tamsulosin	AE rate over study period: **significantly increased with combination therapy (OR 1.59)** Dropout rate: **significantly increased with combination therapy (OR 1.70)** AEs in combination group: myalgia, headache, back pain, pharyngitis, dizziness
Efficacy Review				
Author	Study Design	Number of Participants	Intervention	Findings
Jin 2011	Randomized controlled trial	203	Sildenafil ± doxazosin	IPSS total change: **significantly improved with combination therapy compared to monotherapy (−8.5)** Voiding: not compared Storage: not compared QoL: **significantly improved with combination therapy compared to monotherapy (quantitative value not provided)** Qmax change: not compared PVR change: not compared
Kallidonis 2020	Meta-analysis	Not reported	PDE5i ± α-blocker	IPSS total change: **significantly improved with combination therapy compared to monotherapy (MD −1.72)** Voiding: not compared Storage: not compared QoL: not compared Qmax change: no significant difference between groups PVR change: no significant difference between groups
Kim 2017	Randomized controlled trial	432	Tadalafil ± tamsulosin	IPSS total change: **significantly improved with 0.4 mg tamsulosin/5 mg tadalafil combination therapy compared to monotherapy (−1.32)**, no significant difference with 0.2 mg tamsulosin/5 mg tadalafil combination therapy Voiding: **significantly improved with 0.4 mg/5 mg dose combination therapy (−1.17)** Storage: no significant difference between groups QoL: not compared Qmax change: no significant difference between groups PVR change: no significant difference between groups
Kumar 2014	Randomized controlled trial	75	Tadalafil ± alfuzosin	IPSS total change: **significantly improved with combination therapy compared to monotherapy (−5.9)** Voiding: **significantly improved with combination therapy compared to monotherapy (−3.2)** Storage: **significantly improved with combination therapy compared to monotherapy (−2.7)** QoL: **significantly improved with combination therapy compared to monotherapy (−1.5)** Qmax change: no significant difference between groups (2.5 mL/sec) PVR change: **significantly improved with combination therapy compared to monotherapy (−42.4 mL)**
Sebastianelli 2019	Non-Randomized experimental study	75	Tadalafil ± tamsulosin	IPSS total change: no significant difference between groups (−1.8) Voiding: **significantly improved with combination therapy compared to monotherapy (−1.5)** Storage: no significant difference between groups (0.1) QoL: no significant difference between groups (−0.5) Qmax change: **significantly improved with combination therapy compared to monotherapy (2.0 mL/sec)** PVR change: not compared
Singh 2014	Randomized controlled trial	125	Tadalafil± tamsulosin	IPSS total change: no significant difference between groups (−4.9) Voiding: not compared Storage: not compared QoL: **significantly improved with combination therapy compared to monotherapy (−0.46)** Qmax change: no significant difference between groups (1.03 mL/sec) PVR change: no significant difference between groups (−30.62 mL)
Safety Review				
Author	Study Design	Number of Participants	Intervention	Findings
Sun 2018	Meta-analysis	1052	α-blocker, PDE5i,combination	IPSS total change: **significantly improved with combination therapy compared to monotherapy (WMD 4.19)** Voiding: not compared Storage: not compared QoL: **significantly improved with combination therapy compared to monotherapy (WMD 0.68)** Qmax change: **significantly improved with combination therapy compared to monotherapy (WMD 1.86)** PVR change: **significantly improved with combination therapy compared to monotherapy (WMD −22.58)**
Kai Sun 2020	Meta-analysis	694 for IPSS total and Qmax, 506 for Storage and Voiding, 578 for QoL, 619 for PVR	α-blocker, PDE5i, combination	IPSS total change: **significantly improved with combination therapy compared to monotherapy (MD −3.03)** Voiding: **significantly improved with combination therapy compared to monotherapy (MD −0.98)** Storage: **significantly improved with combination therapy compared to monotherapy (MD −0.66)** QoL: **significantly improved with combination therapy compared to monotherapy (MD −0.33)** Qmax change: **significantly improved with combination therapy compared to monotherapy (MD 1.51)** PVR change: no significant difference between groups (MD −4.05)
Yi Sun 2020	Meta-analysis	2472	α-blocker, PDE5i, combination	IPSS total change: **significantly improved with combination therapy compared to monotherapy (MD −1.30)** Voiding: not compared Storage: not compared QoL: **significantly improved with combination therapy compared to monotherapy (MD −0.15)** Qmax change: **significantly improved with combination therapy compared to monotherapy (MD 1.01)** PVR change: **significantly improved with combination therapy compared to monotherapy (MD −0.54)**
Wang 2014	Meta-analysis	29384	PDE5i, α-blocker, combination	IPSS total change: **significantly improved with combination therapy compared to monotherapy (MD −2.91)** Voiding: no significant difference between groups (MD −1.84) Storage: no significant difference between groups (MD −1.58) QoL: not compared Qmax change: no significant difference between groups (MD 1.50) PVR change: not compared
Wang 2015	Meta-analysis	not reported	PDE5i, α-blocker, combination	IPSS total change: **significantly improved with combination therapy compared to monotherapy (MD −3.97)** Voiding: not compared Storage: not compared QoL: **significantly improved with combination therapy compared to monotherapy (MD −0.81)** Qmax change: **significantly improved with combination therapy compared to monotherapy (MD 2.22)** PVR change: **significantly improved with combination therapy compared to monotherapy (MD −23.43)**
Yan 2014	Meta-analysis	437 for IPSS total, 204 for Qmax	PDE5i, α-blocker, combination	IPSS total change: **significantly improved with combination therapy compared to monotherapy (MD −4.21)** Voiding: not compared Storage: not compared QoL: not compared Qmax change: **significantly improved with combination therapy compared to monotherapy (MD 1.43)** PVR change: not compared
Zhou 2019	Meta-analysis	621 for IPSS total, Qmax, and PVR, 433 for Storage and Voiding, 503 for QoL	Tadalafil ± tamsulosin	IPSS total change: **significantly improved with combination therapy compared to monotherapy (MD −3.21)** Voiding: **significantly improved with combination therapy compared to monotherapy (MD −1.00)** Storage: no significant difference between groups (MD −0.75) QoL: **significantly improved with combination therapy compared to monotherapy (MD −0.36)** Qmax change: **significantly improved with combination therapy compared to monotherapy (MD 0.98)** PVR change: no significant difference between groups (MD −9.02)

AE= Adverse Effects, BPH= Benign Prostatic Hyperplasia, IPSS= International Prostate Symptom Score, IPSS QoL= International Prostate Symptom Score Quality of Life subcategory, PDE5i = Phosphodiesterase Inhibitor, MD= mean difference, OR= odds ratio, WMD= weighted mean difference, SBP= systolic blood pressure, DBP= diastolic blood pressure, Qmax= maximum urine flow rate, PVR= post void residual.

### Efficacy of PDE5i and α-blocker combination vs PDE5i alone

Thirteen studies focused on PDE5i medications as monotherapy vs combination therapy with α-blockers. When compared to PDE5i monotherapy, combination therapy showed additional symptom improvement in most studies. Most studies detected improvement in both IPSS total and IPSS QoL metrics (if reported) for the combination therapy when compared to PDE5i monotherapy with additional changes in IPSS ranging from −1.30 to −8.5 and changes in IPSS QoL ranging from −0.15 to −1.5 [17–26]. Two exceptions were that Sebastianelli et al. and Singh et al. did not show statistically significant improvement in IPSS total, though Singh et al. did show a significant improvement in IPSS QoL of −0.46 and Sebastianelli et al. showed that the combination therapy group had a non-significant numerical improvements compared to PDE5i alone in both scores [19,27]. Many studies reported additional benefit with combination therapy in improving Qmax, PVR, or both when compared to PDE5i monotherapy [18,20–25,27]. Improvements in PVR ranged from −0.54 mL to −42.4 mL [18,21,23,24]. Improvements in Qmax ranged from 0.98 to 2.22 mL/s [20–25,27]. These metrics were not reported in some studies, and four of the studies included both metrics and found no significant difference in either [17,19,28,29].

### Safety of PDE5i and α-blocker combination vs PDE5i alone

All studies on α-blocker and PDE5i combination therapies that reported safety data indicated that combination therapy was safe and well tolerated. Of the 25 studies with sufficient safety data, 7 reported significantly increased incidence of adverse effects in the combination therapy group over the study period [17,20–23,28,30]. These were uniformly mild to moderate in severity, and only Zhou et al. reported an increased study dropout rate from the combination therapy group with an odds ratio of 1.70 [20]. Other studies did not consistently report dropout rates or reasons for dropout, and those that reported this metric did not find it to be statistically significant (Table 1). Importantly, this combination therapy did not significantly increase the risk for symptomatic hypotensive episodes [17–24,26–28,30–37]. Jin et al. measured vital signs including heart rate, systolic blood pressure, and diastolic blood pressure of the patient groups before and after treatment, and the group did not show any significant changes with either the combination therapy or PDE5i alone compared to baseline or to each other [26]. Takeda et al. reported a statistically significant decrease in diastolic blood pressure of 1.6 mmHg and increase in heart rate of 1.4 bpm in the combination therapy group, though this was not determined to be clinically meaningful [38]. The most reported side effects for the combination therapy were the same as for either monotherapy across all studies and included headache, dizziness, dyspepsia, and myalgia.

## Discussion

There is strong evidence for the safety of PDE5i medications in combination with α-blockers to treat the symptoms of LUTS/BPH. Though many studies indicated increased prevalence of mild and moderate adverse effects, which sometimes led to increased study drug discontinuation, they reported that there is no significant increase in serious adverse events, symptomatic hypotension, or change in systolic blood pressure [26,37,38]. Only Takeda et al. reported a decrease in diastolic blood pressure and an increase in heart rate, but the degree of change was not clinically meaningful [38]. No study in this review with combination therapy reported treatment-related dangerous hypotension. Additionally, the adverse events reported were of the same types as those reported in monotherapy treatment groups and of equal severity, though at higher rates in some studies. Overall, current evidence shows that the combination therapy in patients without contraindications to either drug does not have significantly increased risk of serious adverse events, including the feared theoretical risk of compounded hypotension.

Twelve out of 13 studies reviewed showed additional benefits in IPSS total and/or QoL subscores by adding α-blockers when compared to PDE5i alone, though whether this benefit is clinically significant may be less clear. A total IPSS score change of 3 points from baseline has been found to be clinically detectable, a threshold which was shown to be surpassed by Jin et al., Kumar et al., Sun et al. (2018), Kai Sun et al. (2020), Wang et al. (2015), Yan et al., and Zhou et al. [18,20–22,24–26]. Despite statistical improvement, this threshold was not met in Kallidonis et al., Kim et al., Yi Sun et al. (2020), and Wang et al. (2014) [17,23,28,29]. Changes in PVR and Qmax were much less consistently reported, with many studies not assessing these outcomes. Combination therapy shows added benefits in Qmax and PVR improvement over PDE5i drugs alone in many of the studies reviewed, though the heterogeneity of reporting this data precludes drawing wider conclusions. Studies directly comparing PDE5i monotherapies to combination therapy predominantly recommended the combination as most effective in treating LUTS/BPH symptoms and improving IPSS quality of life. This may indicate that the two classes of medication work to alleviate LUTS/BPH symptoms through separate mechanisms and that combining the two can help surpass the maximum effect of either alone. PDE5i medications are postulated to improve LUTS/BPH through a combination of decreased autonomic hyperactivity of the bladder and prostate, increased NO activity in the prostate, and Rho-kinase endothelin inactivation mediated by cGMP [39].

Other combination therapies involving PDE5i medications for LUTS/BPH have also been shown to be safe and effective. For patients with large prostate volume, medications, such as 5-α reductase inhibitors (5ARIs) may be important treatments. For those with residual symptoms after starting 5ARI, severe LUTS/BPH at diagnosis, or ED, combination with PDE5i medications can be beneficial in symptom reduction. In addition, it can provide faster relief before the full benefit from 5ARIs is seen [40]. There is also evidence for the safety and efficacy of combination PDE5i medications with the β-3 agonist mirabegron. This can be considered in patients who are started on PDE5i monotherapy with residual OAB symptoms after full medication effect [41]. PDE5i medications are helpful in treating patients who have comorbid ED and provide multiple opportunities for combination therapies in those who require multiple medication classes to control their LUTS.

This paper is limited by the quantity and quality of available evidence at the time of search. Not all papers communicated the same outcomes, making direct comparisons difficult at times. Because IPSS total score was commonly reported, it was used as a basis for comparing overall symptom improvement. However, other metrics such as IPSS QoL, PVR, and Qmax were discussed when appropriate. Quantitative statistical analysis was not performed in order to allow inclusion of a broader range of studies in this discussion. Additionally, the studies included did not include data from long-term follow-up. This limits discussion of long-term safety and efficacy as well as the need for further intervention.

The findings in this paper have implications for the treatment of patients who do not achieve sufficient symptomatic relief with PDE5i monotherapy. Currently, AUA and EAU guidelines indicate that medical management should be offered to patients before surgical interventions are discussed, and the AUA recommends that surgeries only be offered to patients who have failed medical management due to intolerability or insufficient improvement [12,14]. Without clear and concordant recommendations that PDE5i and α-blocker combination therapy is safe and offers additional benefits compared to PDE5i monotherapy, providers may be hesitant to prescribe this regimen. Instead, they may counsel patients towards surgical management, which is currently done most commonly through transurethral resection of the prostate (TURP), holmium laser enucleation of the prostate (HoLEP), photoselective vaporization of the prostate (PVP), simple prostatectomy, or minimally invasive office-based treatments. Each of these procedures carry risks including serious infection, bleeding, temporary or permanent urinary incontinence, formation of urethral stricture, retrograde ejaculation, and need for re-intervention [14]. The rates of these complications are low but are serious when they occur. These treatments are very effective for symptomatic improvement in LUTS/BPH, though they do not improve erectile function and in many cases are accompanied by new sexual dysfunction. The cost for these procedures are estimated to range from $5,157 for TURP to $8,000 for prostatectomy, though this varies widely based on the institution and details of the individual procedure [42,43]. Costs rise when additional post-intervention treatment or hospital admission is required. The cost of combination therapy with generic tadalafil and generic tamsulosin would be less than $500 for 5 years of daily treatment [44].

## Summary

In the treatment of LUTS/BPH, combination therapy with PDE5i medication and α-blockers was found to be more effective than PDE5i medications alone without a significant increase in serious adverse events, though the improvements seen in objective measures often did not meet established thresholds for clinical significance. Urologists may use this information in shared decision-making with patients who may benefit most from combination therapy. For those with severe symptoms or who do not achieve sufficient reduction with monotherapy, α-blocker and PDE5i combination therapy should be seriously considered, as it has been well tolerated and safe in the populations studied. This regimen may be especially helpful in patients that are not surgical candidates or that have comorbid ED. Further studies should investigate the clinical significance of additional symptom reduction with combination therapy to definitively evaluate whether the addition of an additional medication, which carries some increased risk despite overall safety, is overall worth recommending. As the aging population grows, there will be more patients with multiple comorbid urologic conditions desiring a cheap, safe, convenient, and effective medication regimen, and having clear and concordant recommendations by governing bodies will be important to guide clinical practice.

## Abbreviations list

ED: erectile dysfunction
PDE5i: phosphodiesterase-5 inhibitors
LUTS: lower urinary tract symptoms
BPH: benign prostatic hyperplasia
AUA: American Urological Association
EAU: European Association of Urology
